# Considerations for designing socially assistive robots for older adults

**DOI:** 10.3389/frobt.2025.1622206

**Published:** 2025-10-16

**Authors:** Samuel A. Olatunji, Veronica Falcon, Anjali Ramesh, Wendy A. Rogers

**Affiliations:** College of Applied Health Sciences, University of Illinois Urbana-Champaign, Champaign, IL, United States

**Keywords:** aging, human-robot interaction, domestic robots, user-centered design, technology acceptance

## Abstract

Social robots have the potential to support the health activities of older adults. However, they need to be designed for their specific needs; be accepted by and useful to them; and be integrated into their healthcare ecosystem and care network. We explored the research literature to determine the evidence base to guide design considerations necessary for socially assistive robots (SARs) for older adults in the context of healthcare. We identified various elements of the user-centered design of SARs to meet the needs of older adults within the constraints of a home environment. We emphasized the potential benefits of SARs in empowering older adults and supporting their autonomy for health applications. We identified research gaps and provided a road map for future development and deployment to enhance SAR functionality within digital health systems.

## 1 Introduction

### 1.1 Understanding older adults

The World Health Organization (WHO) estimated that by 2050, the population of individuals aged 60 years and older will double, reaching approximately 2 billion people (WHO, 2024). According to current projections, one in four people living in Europe and North America, by 2025, will be over 65 (United Nations, 2024). At this growth rate, there may likely be older adults by who require support from younger adults who will be insufficient in physical number and resource capacity to manage the care demands (United Nations, 2024). The demographic trend of global aging presents profound challenges and opportunities for healthcare systems (Wheatley, 2024). This demographic shift is not confined to developed nations but is a global phenomenon, with significant implications for health systems across diverse socioeconomic and social landscapes (Officer and de la Fuente-Núñez, 2018).

As the proportion of older adults increases, the demand for healthcare services and applications has to be tailored to address the unique needs of this population, which includes the desire to age with independence (Wheatley, 2024) and remain engaged in their communities (Rogers et al., 2020a). This means living in their home and community safely as well as enjoying their autonomy comfortably, regardless of age, income, or ability level (NIA, 2023). Achieving these goals contributes to health, which encompasses their physical, mental and social wellbeing and not merely the absence of disease or infirmity (Pronk and Krämer, 2021).

The older adult population is diverse, with differences in culture, racial/ethnic background, education, health status, living arrangements, family structures, cognitive capabilities, physical abilities, and personalities (Czaja et al., 2019). Activities of daily living, instrumental activities of daily living, enhanced activities of living, and digital activities of daily living are critical to supporting wellbeing and healthy aging (Katz, 1983; Rogers et al., 1998; Rogers and Mitzner, 2020; Mois and Rogers, 2024). The needs of older adults across these categories vary widely (Czaja et al., 2019), presenting challenges to designing adaptable health systems. Success in addressing these challenges will maximize the opportunities, health benefits, and flourishing of the growing older population (Toure et al., 2012). Therefore, understanding this variability in the demographic spectrum is critical for developing relevant health support devices and systems that address the wide range of older people’s needs to ensure they can enjoy health and age successfully.

### 1.2 Current resources for assistance

Resources for older adults’ needs include support from professional caregivers, family members, and friends. Many older adults rely on their family caregivers to provide support (Rajanala et al., 2020). Often, these caregivers are middle-aged and older women themselves who may also be providing care for their children and other family members. Caregiver burden is associated with a decline in patient healthcare and can negatively impact physical and mental wellbeing of caregivers and patients (Rajanala et al., 2020). There are positive aspects of caregiving such as familial support and sense of purpose (Mishra et al., 2023). Reducing the burdens and enhancing the positive aspects of caregiving are purposes of assistive technologies.

Current assistive technologies include wearable devices, smart home systems, robots, eHealth technologies, and wayfinding technologies (Pramod, 2023). Assistive technology has developed rapidly over recent years; however, these tools are not enough. Although current resources are assumed abundant and continuously advancing, these solutions are often expensive, not geared toward the needs of older adults, or difficult to use by caregivers. Key barriers associated with assistive technology usage include fear of dependence, cost, privacy and security concerns, and autonomy (Albina and Hernandez, 2018). Healthcare workers recognize the benefits of assistive technologies and note that these resources may reduce cost and care provider burden; increase healthcare access; and enhance safety and quality of care (Sommer et al., 2025). The scope of assistive technology is broad and can encompass a wide range of tools. Integrated technologies and artificial intelligence-driven solutions are becoming more prominent and will only advance further over time.

### 1.3 Assistive robots as support for older adults

Robots represent a type of assistive technology with potential to support older adults in a variety of ways. For example, assistive robots can perform tasks to support the needs and abilities of older adults in their everyday lives (Mitzner et al., 2014). One category of assistive robots is mobile manipulators, that interact with the physical world and provide assistance to older adults (Smarr et al., 2014; Beer et al., 2017). Assistive robots offer a wide range of support including cognitive support, which can involve medication reminders, schedule planning, and appointment reminders (Lee and Riek, 2018; Law et al., 2019; Olatunji et al., 2025). Assistive robots can provide physical assistance by supporting older adults in getting dressed, delivering items, reaching high or low places, and removing hazards from the home (Fiorini et al., 2021). Similarly, assistive robots can offer social support by providing a sense of companionship to older adults, facilitating social participation, and encouraging social engagement (Khosla et al., 2021; Collins et al., 2024). The support that assistive robots provide extends beyond the individual users. Assistive robots can support family caregivers and professional care staff by reducing their workload, especially for repetitive or burdensome tasks (Wang et al., 2017; Collins et al., 2025). Many studies conducted to understand older adults’ needs and preferences as they relate to assistive robots were done in the United States (Mitzner et al., 2014; Olatunji et al., 2025; Beer et al., 2017; Lee and Riek, 2018) and Canada (Wang et al., 2017). Studies have also been conducted in Japan (Khosla et al., 2021), and in Australia as well as various European countries including Italy, Spain, the United Kingdom, Germany and the Netherlands, as reviewed in Smarr et al. (2014). Despite the potential of assistive robots as reflected in the development and research studies, there remains a significant gap between the potential of these robots and their acceptance by older adults for long-term use. One reason could be the absence of “socialness” or the feeling of “social connection” between the robot and the older adult. Much of the support that older adults receive comes from friends, family, or professional caregivers, with social interactions being an integral part of the support. For example, if a friend visits an older adult’s home to drop off their prescription, a conversation might start up about the latest TV shows or family news, thereby adding a social component to the support being offered. Consequently, for assistive robots to meet their potential, they may need to encompass the social components and dynamics that an older adult might expect or desire when receiving support.

### 1.4 Evolution of socially assistive robots

**FIGURE 1:**
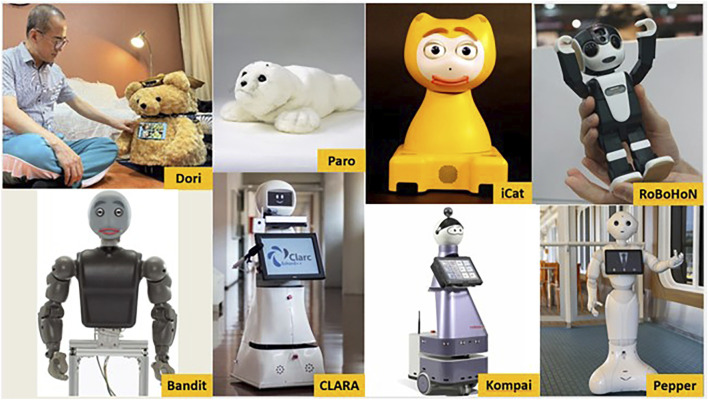
Socially assistive robot examples.

One class of assistive robots is socially assistive robots (SARs), which incorporate social interactions in the assistance they provide (Feil-Seifer and Matarić, 2005). The goal of SARs is to create close and effective interactions with a human user to provide assistance to users and achieve measurable progress in convalescence, rehabilitation, learning, and other daily activities (Feil-Seifer and Matarić, 2005). The “socialness” of SARs is not well defined in the existing literature but has been attributed to the robot’s capability to produce speech and physical gestures and to receive direct input from a user (Feil-Seifer and Matarić, 2005). This definition is limited in that it fails to include crucial elements of social interactions, such as following sociocultural norms, being socially aware of their environment, or displaying social intelligence (i.e., thoughts and feelings; Henschel et al., 2021). Figure 1 presents examples of SARs where such elements have been incorporated. The technological components of SARs has advanced over time. For instance, the emergence of advanced sensors, longer battery life, rapid prototyping, multimodal communication applications, actuation power, generative AI, image processing and pattern recognition capabilities in robots (Rogers et al., 1998; Tapus et al., 2007; Ishak, 2020; Gongor and Tutsoy, 2025) have expanded the capabilities of SARs to support older adults.

Robots with higher levels of socialness may be more accepted and trusted by users, highlighting how their social capabilities influence the user’s thoughts and opinions (Kadylak et al., 2023; Stuck and Rogers, 2018; Van Assche et al., 2024). Users desire streamlined communication with robots, and this includes the robot being able to demonstrate high levels of social awareness and characteristics (De Graaf et al., 2015). People are more willing to view a robot as a companion when they have high expectations of the robot’s lifelikeness (De Graaf et al., 2015). Therefore, we need to expand our understanding of socialness in socially assistive robots to design robots capable of performing two-way social interactions and being capable of sensing the social environment they are in (Matarić and Scassellati, 2016; Collins et al., 2024; Stanojevic et al., 2025).

### 1.5 Methodology for the review

We conducted a focused minireview of the development and testing of SARs in the past 12 years to determine the evidence for design factors that should guide SAR development to meet the support needs of older adults. Our method involved using keyword such as ‘socially assistive robots’ and ‘older adults’ in Google Scholar and the ACM Digital Library. We identified relevant papers focusing on the SARs supporting older adults with evidence of user testing with older adults, and design factors that should guide SAR development. We reviewed a total of 42 papers discussing assistive robots with 21 of those being specifically focused on SARs for older adults with the design factors included. As we reviewed the papers, we employed a theory-driven approach and a data-driven approach. We used theory to identify key themes in the literature that are critical to design such as appearance, adaptability, levels of autonomy, and more. In addition, we included other themes that were prominent in the literature as design considerations. This approach allowed for an in-depth evaluation of relevant literature on SAR development and testing for this population, highlighting key research gaps, design factors, and recommendations.

## 2 Design considerations for SARs supporting older adults

The outcome of our review revealed the current landscape of SARs supporting older adults. We present this in form of design considerations to help understand the gaps prevalent in designing SARs to support older adults and design considerations that need to be made in the design. We organized the considerations into the following categories: appearance; adaptability and personalization; levels of autonomy; modes of communication; ease of use; and robot functions and abilities.

### 2.1 Appearance

The appearance of an SAR can impact a user’s acceptance, expectations, and trust towards the robot (Pino et al., 2015; Stuck and Rogers, 2018; Langer et al., 2019; Liberman Pincu et al., 2023). The components of a robot’s appearance include its body structure, outline, texture, features, colors, and level of anthropomorphism (i.e., how human-like it looks). When designing SARs, the robot’s appearance should reflect its functionality and abilities (Kwak, 2014). This encourages appropriate expectations from users and reduces possible misattributions of the robot’s abilities or confusion over its function (Kwon et al., 2016; Phillips et al., 2017). An SAR’s social capability, or its socialness, should be conveyed in its design (Bradwell et al., 2021). For instance, incorporating elements such as a face, mouth, and eyes conveys the robot’s abilities for social communication, which can increase a user’s perceived enjoyment when using the robot, further growing their intention to use it (Bradwell et al., 2021). The appearance of SARs should follow cultural expectations and norms based on their intended use and the context of the interactions (Lim et al., 2021; Mois et al., 2023). For example, SARs that incorporate an a-shaped frame, rounded edges, and a mainly white exterior might convey medical or health-related support and abilities (Liberman Pincu et al., 2023). Similarly, robots that offer emotional support or comfort can benefit from a soft or plush-like texture (Nestorov et al., 2014). And robots with faces are often perceived more positively than those without (Ghazali et al., 2018).

These examples are not universal–users’ preferences for the appearance of robots vary depending on the individual’s age, gender, and previous experience with robots (Smarr et al., 2014; Pino et al., 2015). For instance, making a robot look too human-like can have the unintended consequence of causing discomfort or fear (Der Pütten and Krämer, 2014). In addition, appearance preferences may be related to the tasks that the robot is intended to perform, e.g., (Prakash and Rogers, 2015). Moreover, gender-based tasks vary across cultures, which may impact how a user accepts the robot. Cultural context significantly influences appearance preferences and perceptions of socially assistive robots (Papadopoulos and Koulouglioti, 2018).

Cultural diversity and considerations in the design and evaluation of SARs have a significant influence on the user’s perception of, and interaction with the robot (Papadopoulos and Koulouglioti, 2018; Lee et al., 2023). For instance, Bartneck (2008) reported that U.S. participants preferred human-like robots more than the Japanese participants did. Similarly, Kamide and Arai (2017) found that U.S. participants were more comfortable towards human-like robots than were Japanese participants. In contrast, Lee and Sabanović (2014) found that South Korean participants preferred human-like robots and thought they could be part of social life, whereas U.S. participants preferred machine-like robots, and thought of them as tools. These mixed results demonstrate the variability in preferences and highlight the need for further studies to understand cultural impact on robot acceptance. A robot’s features, such as language and communication style, influence the perceptions of people from different cultural backgrounds (Korn et al., 2021). Trovato et al. (2013) found that when the robot greeted the person in his or her own language and in a culturally appropriate way (e.g., bow for Japanese), it was more accepted and liked because it felt close to the person’s culture.

Liberman Pincu et al. (2023) recommended that robot designers begin by focusing on the visual qualities of the robot. These include user preferences (e.g., body structure, outline, and color scheme), and cultural context (e.g., gesture style, gender presentation, color choice). Designers should first consider the context where the SAR will be used, its support, and how its appearance may impact the interaction (Collins et al., 2025).

### 2.2 Adaptability and personalization

Adaptability and personalization in the design of SARs refer to the ability of robots to adapt to the user’s needs and wishes (Pnevmatikos et al., 2022). Moreover, the robot design should offer the opportunity for users to personalize the robots to their unique needs, preferences, and capabilities (Langer et al., 2019; Pnevmatikos et al., 2022). Personalization can include the general design of the robot (e.g., appearance, voice, gender), its behavior, the services it offers, or its social capabilities (Pino et al., 2015). Offering users personalization options can positively impact their acceptance of SARs (Pino et al., 2015; Liberman-Pincu and Oron-Gilad, 2022; Hofstede et al., 2025). Supporting users in customizing the robot according to their preferences can make the interaction more personal (Pino et al., 2015) and potentially mitigate issues of broad generalizations when designing robots for older adults (Sparrow and Sparrow, 2006; Sharkey and Sharkey, 2010).

Personalization can be done in areas such as the robot’s voice, speed, language, color, and level of language complexity. Additionally, SARs can have varying levels of socialness, which can be adapted to the user’s preferences, the context of the interactions, and cultural expectations (Robinson and Nejat, 2022). Another level of adaptability that SARs can offer is the level of support they offer to users and what tasks they perform (Kellmeyer et al., 2018). As the needs of older adults change over time, the robot could adapt to their changing needs to offer more appropriate and personalized support (Kellmeyer et al., 2018).

### 2.3 Levels of autonomy

Levels of autonomy (LOA) can be explained as the degree to which a robot would carry out certain functions in its defined role of assisting the user (Beer et al., 2014). Lower LOA involves the user manually controlling the robot’s actions whereas higher LOA, involves the robot autonomously carrying out its actions with minimal or no human input (Beer et al., 2014). Other forms of ‘medium’ LOAs exist that can be considered in the context of user consent and/or exception. An example is the human-oriented semiautonomous level wherein the user must explicitly agree to suggested activities before the robot carries them out (Steinfeld et al., 2006). This usually supports users’ awareness of and control over the robot’s behavior but comes at the cost of increased communication demands. Another example is the robot-oriented semiautonomous level, wherein the robot informs the user as it initiates and implements actions unless the user objects (Steinfeld et al., 2006). This generally implies less communication/instruction demands for users but could inadvertently impose a perceived risk of taking over the autonomy of the user (Johnston, 2022). These concerns have driven LOA development to incorporate a continuum of levels that prioritizes independence, and a sense of control, without diminishing robot support needed in specific tasks. Beer, Fisk and Rogers (2014) developed a framework that could help researchers identify composites of these levels, along with their accompanying costs, benefits, and limitations (Beer et al., 2014).

Adjusting the robot’s involvement in a variety of tasks can facilitate their use (Kaber, 2018). In the context of robot-assisted care of older adults, it is important to factor in the independence of the older adults, the variety of situations and tasks involved, and the quality of the interaction (Flemisch et al., 2011). One approach is a condensed version of the autonomy levels such as two LOA modes suitable for assistive robots supporting older adults in specific utilitarian tasks–a low LOA mode and a high LOA mode wherein both kept the human in the loop (Olatunji et al., 2021). These models were tested under different conditions to evaluate their suitability and influence in the interactions. The older adults were able to effectively accomplish the defined tasks using both LOA modes and generally preferred an LOA mode that facilitates active involvement in the tasks.

It is pertinent to ensure that older adults retain control and active participation in everyday activities while enjoying support of the robot as needed. Therefore, to preserve the autonomy of older adults and to facilitate robot support, we propose aiming for user-centered autonomy wherein the LOA is adaptable to the user. The goal would be to identify a feasible design path for the SARs to adapt its autonomy level to support the needs and preferences of the older adults in maintaining their independence.

### 2.4 Modes of communication

Studies have evaluated communication modes for SARs supporting older adults in the areas of physical support (Fischinger et al., 2016; Gross et al., 2017), rehabilitation (Ye et al., 2020), social interaction (Fong et al., 2003), cognitive support (Gross et al., 2017; Góngora Alonso et al., 2018), and safety monitoring (Kuo et al., 2013). Insights from these studies highlight the merits of voice feedback combined with visual feedback. They pointed to the effectiveness of continuous feedback over discrete feedback to keep users constantly aware of the state of the interaction.

Human environments are complex and nuanced and, as such, require flexible communication. Nonverbal behaviors, speech, and communication style can be perceived differently across cultures. In countries such as China, where implicit form of communication is more common, participants responded more positively when this form of communication was present in their interactions (Papadopoulos and Koulouglioti, 2018).

Despite speech being a common mode of communication, this may not be appropriate in all contexts. Other modes of communication that have been explored include haptics, facial and emotional recognition, and gestures. Affective touch, haptics, and mimicry has been studied in SARs and, although interactions can be positive, there were significant engineering and ethical challenges at play (Paterson, 2023). Facial and emotional recognition within SARs have been employed, but recognition is simply the first step. Further studies must be conducted to improve emotional interactions and natural human exchanges, while ensuring comfortable interactions (Ruiz-Garcia et al., 2018). Moreover, additional research regarding how older adults interpret the emotion displayed by a robot is needed; early studies suggested that older adults may misidentify a robot’s emotions, which can lead to communication failures (e.g. (Beer et al., 2015)).

Multimodal communication has been explored in a case study with a quadriplegic and mute individual, who utilized a PR2 robot with a layered communication system instead of solely relying on speech (Chen et al., 2011). The success of this approach illustrated the value of designing assistive robots that accommodate diverse user needs (Chen et al., 2011).

An aspect of communication that still requires further work is evaluating transparency-related feedback content in communication that touches on aspects such as the mode and timing of the feedback provided by the robot (Markfeld et al., 2021). We recommend developing and evaluating user-centered communication that considers appropriate feedback content, relevant modalities, and suitable timing options to enhance transparency in the operation of the SARs supporting older adults. It is critical to implement the required transparency in communication for different SAR platforms, tasks, and situations that match the needs of the older adults in a home healthcare context.

### 2.5 Ease of use

Emphasis on ease of use regarding SARs is crucial and can allow for the development of an effective tool that accounts for varying and diverse populations, such as older adults. Utilizing natural interaction, multiple modalities of communication, and context aware design can significantly foster positive interaction (Fong et al., 2003; Nestorov et al., 2014). Convenience of the user and perceived enjoyment play a role in acceptance of SARs with older adults (Luo et al., 2024). This suggests that older adults are more likely to accept SARs when they are enjoyable and can seamlessly fit into their everyday lives. Cognitive and perceptual factors should be taken into consideration when considering how to ensure ease of use. To design for the unique needs of the older adults, simplicity, convenience, and usefulness is key (Zafrani et al., 2023).

Studies have explored common human robot interaction principles and highlighted key aspects of ensuring ease of use (Heerink et al., 2010); however, SARs may be designed based on developers’ assumption of older adults’ opinions (Søraa et al., 2023). Additionally, research often focuses on short term usability testing, making it difficult to know how ease of use translates over an extended period of time (Beer et al., 2017). As such, gaps in research regarding ease of use for SARs include over reliance on short term testing, increased complexity of SARs due to assumptions, and simplification of ease of use. We recommend involving older adults’ more during development and testing phases and pushing past preconceived notions of what designers believe older adults need. Focusing on simple and natural interactions will improve ease of use and allow SARs to be geared towards the older adult population. Participatory design methods can enable older adults to provide inputs based on their lived experiences (Rogers et al., 2022; Olatunji et al., 2024).

### 2.6 Robot functions and abilities

The functions of SARs differ by the type and context of use. In general, it is beneficial for the robot to possess interaction intelligence. For robots to provide effective personalized care for older adults, context awareness and the ability to communicate that awareness to the care recipient is key (Nestorov et al., 2014). This awareness is expected to guide the actions of the robot in providing care (Johansson-Pajala et al., 2020).

The awareness capabilities of SARs could be further enhanced by incorporating natural language processing, facial expression, emotional and body language recognition (Matarić and Scassellati, 2016). This enhances the quality of care that SARs can provide. For instance, these capabilities are relevant in understanding the emotions or body language of the care recipient and adapting response strategies or level of care as needed based on changes observed (Góngora Alonso et al., 2018). As these capabilities advance, it is critical to consider the risk of the person developing an excessive emotional bond to the robot (Johnston, 2022), alongside privacy consideration for the user (Collins et al., 2025). There is the need to better understand the implications and how to leverage these awareness capabilities to provide more comprehensive emotional support and regulation.

In designing robot functions and abilities, it is crucial to keep it goal-oriented (i.e., to support specific tasks or activities of daily living that match the needs of the older adult). Older adults are more likely to accept or adopt an SAR if they perceive its usefulness (Beer et al., 2017; Olatunji et al., 2025). SARs should be aware of the specifics of task details such as time required for completion, constraints connected to the task, demands and dependencies in the task, requirements for the task, and progress in the task (Chen and Barnes, 2014; Hoffman, 2019).

It is beneficial for SARs to have the capability to be integrated with existing home healthcare support systems such as digital voice assistants, smart home hubs, and remote sensing or monitoring systems that provide data needed to inform personalized care (Dahl and Boulos, 2014; Yang et al., 2020). We understand that there are inherent risk of security breaches and hacking (Poulsen et al., 2020). There are concerns over continuous monitoring and invasion of privacy deterring the older adults from freely expressing themselves or sharing personal information (Davis et al., 2017). These issues and concerns need to be addressed through deliberate design to preserve privacy, ensure security and to provide reassurance needed while using these devices (Probst et al., 2024). This integration has the potential to enhance the robot functions such that it can provide more holistic support, augment the effort of the care network in providing comprehensive health support routines around the home (Mois et al., 2023; Irshad et al., 2025).

## 3 Research roadmap

**FIGURE 2:**
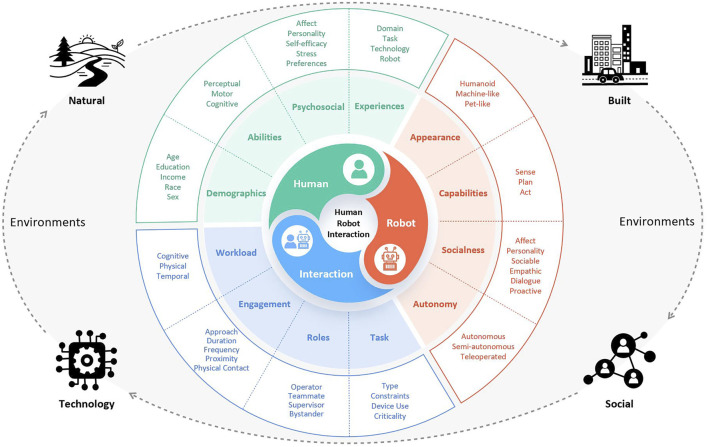
Human-robot interaction framework from Rogers and Mitzner (2026).

In the previous section, we examined the current landscape of SARs to understand the prevailing challenges in designing SARs to support older adults’ autonomy for health applications. We also provided guide design considerations based on the evidence available for the successful design and implementation of SARs to support home health applications for older adults. We are aware that these guidelines are not exhaustive, but they do provide necessary considerations based on the research evidence available during this review. To support understanding of other design considerations that may not have been discussed, we recommend consulting the human-robot interaction framework (Figure 2) developed by (Rogers and Mitzner, 2026).

### 3.1 Human-robot interaction framework to guide design

The Rogers and Mitzner (2026) holistic framework presented the main dimensions of human-robot interaction (HRI): the human, the robot, the interaction, and the environment. The framework can help identify components within the main HRI dimensions that should be considered in the design of SARs for older adults, which the current state of design and practice may not have factored in yet. This can, therefore, be a general guide for design priorities and specifications of SARs supporting older adults within digital health systems.

We highlight design elements from this framework and from the insights gained from our mini review to provide a roadmap for future designs of SARs for older adults in home healthcare contexts: These include.

#### 3.1.1 Human-related elements

SARs should be designed to be aware of the users’ characteristics, preferences, needs, and limitations. For instance, the SARs’ awareness of the user’s physical state (e.g., upper or lower body physical limitations, range of movement), cognitive state (e.g., memory or decision-making abilities), emotional state or mood (e.g., happiness, fear) helps the robot adapt its support to match the needs and abilities of the care user. The robot should be able to process information regarding the workload or stress the human is experiencing and respond/adapt as needed (HasanPour et al., 2023).

#### 3.1.2 Robot-related elements

Robot elements that impact the perceptions and willingness to use the robot should be incorporated in the design such as appearance, form factor, functions of the robot, as well as the ability of the robot to process information pertaining to its behavior and operation. For instance, the SAR should be able to communicate information on the steps it is taking in carrying out tasks, its degree of reliability in carrying out tasks, its current degree of autonomy and other information that will support the older adult’s understanding of the interaction. User-friendly instructional support materials to guide the use of the SAR are an additional requirement that should not be overlooked (Conati and VanLehn, 2001; Tsai et al., 2012).

#### 3.1.3 Interaction-related elements

Interaction-related elements (such as the task, interaction roles, engagement and workload) influence the interaction between the older adult and the SAR and should be incorporated into the design. Examples of such elements include awareness and dynamics of the teamwork (Sparrow and Sparrow, 2006; Dahl and Boulos, 2014; Tsai et al., 2022; Liberman Pincu et al., 2024). This includes understanding task allocation as the older adult and robot work together within the human-robot autonomy model and how each role will be executed to support the care recipient.

#### 3.1.4 Environment-related elements

Elements such as the type of environment (e.g., indoors, outdoors, corridor, open space), conditions prevalent in the environment (e.g., illumination, clutter, obstacles, weather), environmental constraints, and safety-related environmental information (Chen and Barnes, 2014; Adamides et al., 2017; Honig and Oron-Gilad, 2018) are essential to consider when designing a SAR supporting an older adult in a home healthcare context.

These elements provide crucial next steps that practitioners and researchers can leverage to advance the design of the next-generation of SARs that will truly meet the needs of older adults in different home healthcare contexts. These considerations inform future directions for research, practice, and implementation.

### 3.2 Concluding thoughts

SARs can be designed to enhance the autonomy of older adults in multiple ways. Through the user-centered guide in this article, we provided considerations for designing SARs to meet the needs of older adults within the context of a home environment. These considerations are crucial to integrating SARs with broader digital health systems to enhance overall health outcomes and quality of life for older adults. However, they are only the first step. Continued engagement in the development process with representative older adults in common contexts performing high-need tasks is crucial for successful development of SARs to support older adults.
